# Behaviour and attitudes in HIV (BEAHIV): a national survey study to examine the level of agreement between physicians and patients in symptom reporting

**DOI:** 10.1186/1758-2652-13-S4-P106

**Published:** 2010-11-08

**Authors:** A Rachlis, J Gill, M Harris, J Macleod, C Worthington, J Brunetta, A Tsang, H Hew, J Leith, F Camacho, D Turner, C Fraser

**Affiliations:** 1 Sunnybrook Health Sciences Centre, Toronto, Canada; 2 Southern Alberta Clinic, Sheldon M Chumir Health Centre, Calgary, Canada; 3 Providence Health Care, Vancouver, Canada;; 4 Toronto, Toronto, Canada; 5 University of Calgary, Faculty of Social Work, Calgary, Canada; 6 Maple Leaf Medical Clinic, Toronto, Canada; 7 Tibotec, Medical Affairs, Toronto, Canada; 8 Tibotec, Toronto, Canada;; 9 Damos Inc, Toronto, Canada; 10 Ispos Reid Inc, Montreal, Canada; 11 Cool Aid Community Health Centre, Victoria, Canada

## Background

Management of antiretroviral (ARV)-related symptoms is a major challenge in the treatment of HIV infection, and uncensored reporting by the patient and subsequent acknowledgement by the physician are critical. The primary objective of BEAHIV was to examine the level of agreement between patients and their physicians regarding the presence or absence of 22 symptoms as reported on the HIV Symptoms Distress Module (SDM). P>A non-interventional, observational, cross-sectional survey study was conducted Sept-Nov 2009 across 17 Canadian sites. Data was collected from consenting adult HIV-positive outpatients and their HIV-treating physicians at a single clinic visit. Major inclusion criteria included ability to read and write in English or French.

## Results

1000 patient and corresponding physician surveys were collected. Physician respondents (68% male) had been treating HIV patients for an average of 15 years. 88% of patient respondents were male, 84% were currently on ARVs, 59% had received ARVs >5 years, and 31% had detectable viral load at survey completion. Median age was 46 years, median time since HIV diagnosis was 11 years and median CD4 count was 460 cells/mm3. Fifty-six percent had comorbid conditions (29.5% mental health issues, 18.7% HBV/HCV co-infection, 18.9% metabolic problems), and 72% were taking non-ARV medications. Median total SDM score (out of 84) was 31.0 reported by patients versus 8.0 by physicians. All symptoms, including those most bothersome to patients, were reported more frequently by patients than physicians; symptoms with the largest discordance were trouble remembering (60.2% vs. 16.4%), sexual problems (59.1% vs. 16.4%) and bloating pain/gas (54.3% vs.12.6%).

## Conclusions

This large, Canadian, cross-sectional survey study identified substantial and relevant differences in agreement between HIV patients and their physicians regarding the presence or absence of a defined set of common symptoms associated with HIV and its treatment. Relative to their patients, physicians consistently under-reported patients' symptoms.

